# Purtscher-Like Retinopathy

**DOI:** 10.1155/2015/421329

**Published:** 2015-01-06

**Authors:** Rita Massa, Carolina Vale, Mafalda Macedo, Maria João Furtado, Miguel Gomes, Miguel Lume, Angelina Meireles

**Affiliations:** Department of Ophthalmology, Hospital de Santo António, Centro Hospitalar do Porto, Largo Professor Abel Salazar, 4099-001 Porto, Portugal

## Abstract

Purtscher-like retinopathy is associated with retinal hemorrhages and ischaemia probably due to the complement-mediated leukoembolization. It is a rare and severe angiopathy found in conditions such as acute pancreatitis. *Case*. We present a case of a 53-year-old man who presented with a Purtscher-like retinopathy associated with the development of acute pancreatitis in the context of a Klatskin tumour (a hilar cholangiocarcinoma). The ophthalmologic evaluation revealed the best corrected visual acuity (BCVA) of 20/32 in the right eye (RE) and of 20/40 in the left eye (LE); biomicroscopy of anterior segment showed scleral icterus and fundoscopy revealed peripapillary cotton-wool spots, optic disc edema, and RPE hypo- and hyperpigmentation in the middle peripheral retina in both eyes with an intraretinal hemorrhage in the LE. 15 months after the initial presentation, without ophthalmological treatment, there was an improvement of BCVA to 20/20 in both eyes and optical coherence tomography (OCT) revealed areas of reduction of retinal nerve fiber layer thickness corresponding to the previous cotton-wool spots. *Conclusion*. Purtscher-like retinopathy should not be neglected in complex clinical contexts. Its unclear pathophysiology determines an uncertain treatment strategy, but a meticulous follow-up is compulsory in order to avoid its severe complications.

## 1. Introduction

Purtscher's retinopathy or* angiopathia retinae traumatica* was first described in 1910, by Otmar Purtscher, an Austrian ophthalmologist, in a middle-aged man who complained about bilateral vision loss some hours after a severe head trauma. The ophthalmoscopic examination revealed multiple areas of retinal whitening (*Purtscher flecken*) with intraretinal hemorrhages confined to the posterior pole in both eyes. Despite the gravity of vision loss, the patient recovered his visual acuity without specific treatment [1, 2].

After its original description, this typical fundus appearance was also observed in severe but nontraumatic conditions such as acute pancreatitis, amniotic fluid embolism, and collagen-vascular disorders and was designated as Purtscher-like retinopathy [3, 4].

Some authors estimated an incidence of 0.24 cases per million per year (including Purtscher and Purtscher-like retinopathy), but it is possible that this number can be higher due to some asymptomatic cases [5]. However, it is still considered a rare condition.

We report a case of Purtscher-like retinopathy in the context of acute pancreatitis secondary to a Klatskin tumour (a hilar cholangiocarcinoma). To our knowledge, this is the first case report describing a Purtscher-like retinopathy in a patient suffering from a Klatskin tumour.

## 2. Case Report

A 53-year-old man with multiple cardiovascular risk factors (diabetes, arterial hypertension, dyslipidemia, and obesity) was admitted to the emergency department with jaundice, nausea, choluria, and itch present for three weeks. Abdominal pain and fever were not present. Physical examination revealed jaundice with scleral icterus. Blood tests showed an elevation of liver enzymes (aspartate aminotransferase = 70 units per liter (U/L), alanine aminotransferase = 226 U/L, alkaline phosphatase = 379 U/L, and gamma glutamyltransferase = 3118 U/L) and hyperbilirubinemia (9.16 milligrams per decilitre (mg/dL)). Further investigation was performed and magnetic resonance cholangiopancreatography suggested the presence of a Klatskin tumour (a hilar cholangiocarcinoma) causing a principal bile duct stenosis. Secondary severe pancreatitis developed and by that time, the patient reported bilateral progressive visual loss and floaters.

The ophthalmologic evaluation revealed a best corrected visual acuity (BCVA) with Snellen chart of 20/32 in the right eye (RE) and 20/40 in the left eye (LE). Biomicroscopy of the anterior segment showed scleral icterus without* rubeosis iridis*.

Mydriatic fundoscopy revealed peripapillary cotton-wool spots, optic disc edema, and RPE hypo- and hyperpigmentation in the middle peripheral retina of both eyes. An intraretinal hemorrhage in the left inferior temporal arcade was also seen. Signs of macular edema were not present.

**Figure 1:**
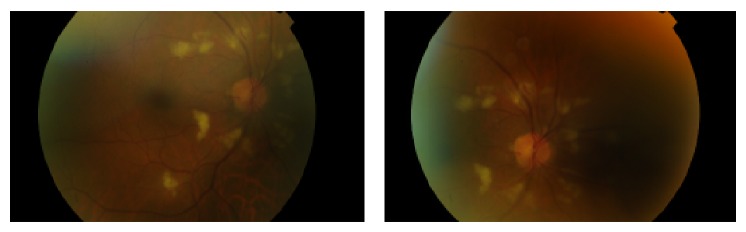
Retinography of the right eye at first ophthalmologic evaluation showing the presence of peripapillary cotton-wool spots and optic disc edema.

**Figure 2:**
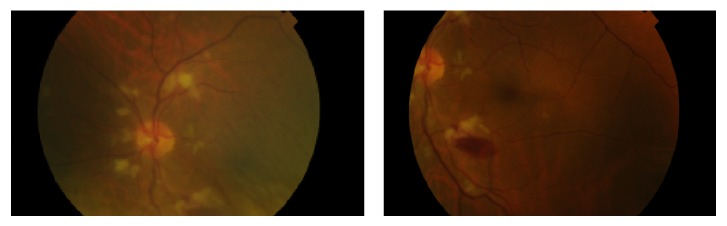
Retinography of the left eye at first ophthalmologic evaluation showing the presence of peripapillary cotton-wool spots, optic disc edema, and an intraretinal hemorrhage in the left inferior temporal arcade.

Due to the patient's severe renal compromise, it was not possible to perform a fluorescein angiography; instead, retinography was carried out at first ophthalmologic evaluation (Figures 1 and 2).

The patient initiated treatment for acute pancreatitis (steroids were not used). Klatskin tumour was considered unresectable and it was decided to initiate palliative chemotherapy with cisplatin and gemcitabine. Due to the severely compromised immune system of the patient and the natural history of Purtscher-like retinopathy, it was decided together with the oncology group not to treat the patient with high-dose steroids.

Five months later, during an ophthalmologic evaluation, the patient reported an improvement in his visual acuity. His BCVA with Snellen chart was 20/20 in both eyes. Biomicroscopy of anterior segment showed anicteric sclerae, without any other alterations.

**Figure 3:**
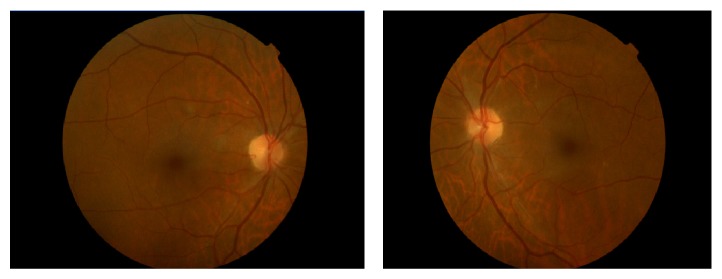
Retinography of the right and left eyes five months after presentation of the retinopathy revealing the presence of only peripapillary soft exudates in the right eye and no significant alterations in the left eye.

**Figure 4:**
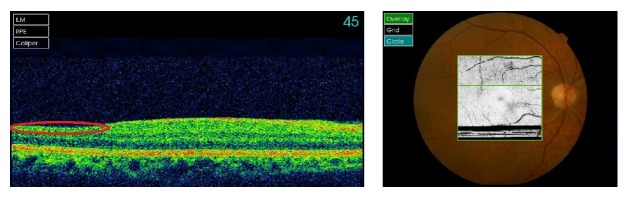
OCT of the right eye five months after presentation of the retinopathy. The red circle reveals the area of reduction of retinal nerve fiber layer thickness corresponding to the previous cotton-wool spots.

**Figure 5:**
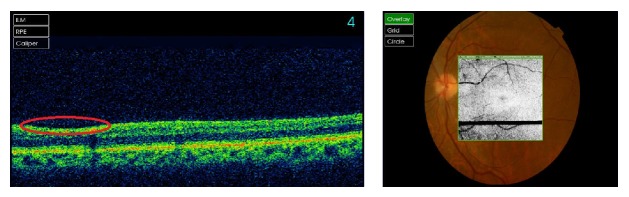
OCT of the left eye five months after presentation of the retinopathy. The red circle reveals the area of reduction of retinal nerve fiber layer thickness corresponding to the previous cotton-wool spots.

Mydriatic fundoscopy revealed only peripapillary soft exudates in the RE and no significant alterations in the LE (Figure 3). Optical coherence tomography revealed areas of reduction of retinal nerve fiber layer thickness in both eyes corresponding to the previous cotton-wool spots (Figures 4 and 5).

**Figure 6:**
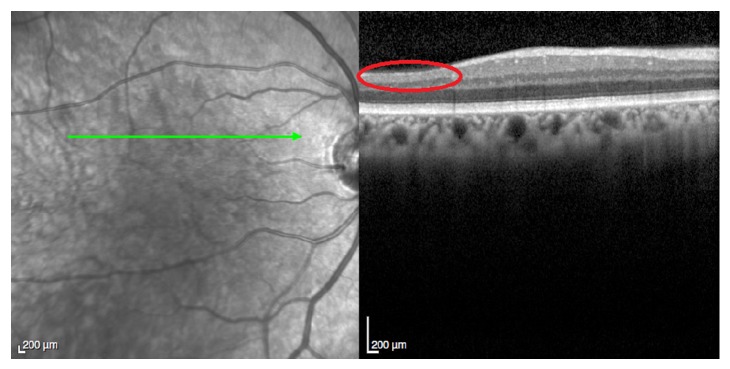
OCT of the right eye fifteen months after presentation of the retinopathy. The red circle reveals the area of reduction of retinal nerve fiber layer thickness corresponding to the previous cotton-wool spots.

**Figure 7:**
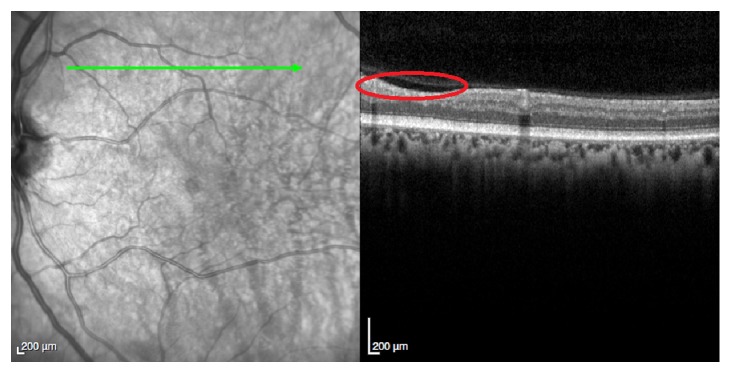
OCT of the left eye fifteen months after presentation of the retinopathy. The red circle reveals the area of reduction of retinal nerve fiber layer thickness corresponding to the previous cotton-wool spots.

In the last visit, 15 months after presentation of the retinopathy, the patient was still under palliative chemotherapy and had no visual complaints and the ophthalmologic evaluation was quite similar to the previous one. Optical coherence tomography revealed areas of reduction of retinal nerve fiber layer thickness in both eyes corresponding to the previous cotton-wool spots (Figures 6 and 7). One month later, the patient died.

## 3. Discussion

Purtscher-like retinopathy associated with acute pancreatitis was first described in 1975 by Inkeles and Walsh [6]. Its development is independent of the severity of pancreatitis, and it has a wide range of manifestations and requires a close follow-up [7].

Purtscher-like retinopathy is a rare and severe angiopathy that begins within some hours to some days after the onset of the systemic disease and is characterized by confluent cotton-wool spots in the posterior pole, few intraretinal hemorrhages, and* Purtscher flecken* in the acute phase.* Purtscher flecken* are polygonal areas of whitening in the inner retina between the retinal arterioles and venules with a characteristic clear zone between the affected retina and an adjacent arteriole, extending for an average of 50 *μ* on either side of retinal arteries and precapillary arterioles. These lesions are pathognomonic of the disease but are present in only half of the cases and must be differentiated from cotton-wool spots which are superficially over vessels and have ill-defined borders [8, 9]. Optic disc swelling as well as retinal edema and pseudo-cherry spot (if macula is affected) can also be observed in the initial phase but are less common. The disease is bilateral in the majority of the cases [2].

The diagnosis is clinical with sudden loss of visual acuity associated with typical fundus appearance in the context of a systemic illness such as acute pancreatitis or long bone fracture.

The most important differential diagnoses to consider include branch or central retinal artery occlusion, hypertensive retinopathy, diabetic retinopathy, and HIV retinopathy with cotton-wool spots [2].

The pathophysiology of Purtscher-like retinopathy remains unclear although some theories have been proposed according to the underlying systemic disease, but it is thought to be caused by embolization (leukocyte aggregates, fat, fibrin, and platelets) that will cause arteriolar occlusion and subsequently ischemia. In the particular case of Purtscher-like retinopathy associated with acute pancreatitis, it has been proposed that pancreatic damage causes release of proteolytic enzymes into the systemic circulation with subsequent activation of complement cascade and the formation of C5a-induced leukocyte, platelet, and fibrin aggregate that will cause retinal embolization and ischemia, but the true mechanism has not been established yet [10–12].

The treatment of the ocular complications has not been proven and their prognosis depends on the affected retinal areas which should be carefully monitored.

In the majority of the cases, the acute lesions resolve spontaneously within 1–3 months after the onset. Nevertheless, there are some reports of successful treatment with intravenously high-dose steroids. In this case report, the improvement of acute pancreatitis was associated with the resolution of the acute ocular lesions and a positive impact on the visual acuity. In fact, the patient underwent palliative chemotherapy with cisplatin and gemcitabine and consequently steroids were never used due to chemotherapy-induced immunosuppression and associated high risk of infection. The decision not to treat the patient's ophthalmological condition was taken after thorough reflection as it could imply extensive retinal neovascularization with involvement of the anterior segment and ultimately neovascular glaucoma with permanent visual loss.

There are no consensual guidelines of the therapeutic approach of this condition [13, 14].

The management of the ocular complications can be particularly difficult due to the “life-threatening” clinical context.

Fluorescein angiography can show capillary nonperfusion, retinal and/or disc edema, perivascular staining, and late leakage from injured retinal vessels providing further information about the severity of the disease. In our case, fluorescein angiography was not performed due to the patient's severe renal compromise; instead macular and papillary optical coherence tomography were used to complement clinical evaluation [2, 3].

Other ancillary exams such as optical coherence tomography, visual field tests, and electrophysiology can also complement the follow-up of the patient [2, 3].

Optic disc pallor can be observed in the late phase of this condition, but it was not verified in this case; atrophy of retinal nerve fiber layer can also occur in the late phase and was observed in our case [2].

Our case illustrates the natural history of Purtscher-like retinopathy which should not be ignored in complex clinical contexts even when there is no history of trauma. Its unclear pathophysiology makes the establishment of a clear treatment guideline more difficult. Nevertheless, a meticulous “follow-up” is mandatory in order to avoid its severe complications.
